# Prevalence of Infection in a Population Can Shape Parasite Virulence

**DOI:** 10.1371/journal.pbio.0030295

**Published:** 2005-07-19

If necessity is the mother of invention, the coevolutionary arms race is the mother of adaptation. For parasites and hosts engaged in an ongoing battle to gain advantage, those adaptations take many forms. A host continually tinkers with resistance mechanisms while the parasite adjusts its means of infection. But if infection causes too much damage to its host, the parasite could destroy its chances of transmission and, thus, survival. Thus, from the parasite's point of view, natural selection must create an optimal balance between the costs of parasitic infection (or virulence)—host death—and the benefits—eluding host defenses and establishing an infection.

Stephanie Bedhomme, Yannis Michalakis, and their colleagues study the coevolution of parasite virulence and host life history traits. In a new study, the researchers modify the standard approach to studying the costs of infection (comparing infected and uninfected groups) by introducing another variable: intraspecific competition between hosts. Specifically, they ask, does infection affect competitive interactions between individuals? As expected, the authors find that infected individuals pay a cost compared to their healthy counterparts. But surprisingly, both infected and uninfected individuals do better when their competitor is infected: parasite costs depend on the infection status of the competitors.

To study the interplay between parasitism and intraspecific competition, Bedhomme et al. worked with the yellow fever mosquito Aedes aegypti and its natural enemy, the single-celled parasite Vavraia culicis. In the wild, mosquitoes get V. culicis infections when larvae ingest spores of the parasite, which enter larval gut cells, where they develop and reproduce. Because the spores need water to survive, it's thought that successful transmission depends on larvae, which live in water, and on two host life history traits: larval developmental time and probability of emergence of the host. [Fig pbio-0030295-g001]

**Figure pbio-0030295-g001:**
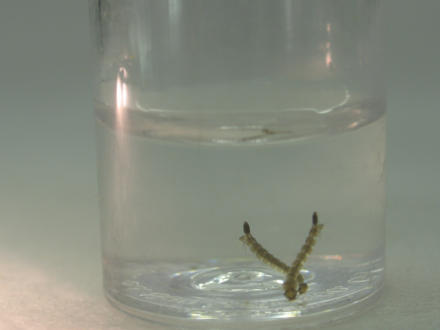
By accounting for competition between infected and uninfected mosquitoes, researchers discovered a link between parasitic virulence and the prevalence of infection in a population

To study this process in the lab, Bedhomme et al. divided recently hatched mosquito larvae into groups of 60 larvae, and exposed half of the groups to the parasite. Larvae were then placed two by two into vials. Vials contained either two uninfected larvae, two infected larvae, or one infected and one uninfected individual.

Larvae were treated with high- and low-food diets. Competitive performance was measured by the probability of reaching adulthood and the time to develop. On the high-food diet, infected and uninfected larvae mostly grew normally and reached adulthood. On a low-food diet, however, infected larvae grew slowly and were less likely to reach adulthood—probably because the parasite had more time to kill them. Being paired with an infected versus uninfected partner did not influence this outcome.

As for time to develop, infected pairs took longer to develop than uninfected pairs, as expected. But with infected and uninfected pairs, infected larvae took longer to develop than their healthy partners, meaning they should be more likely to succumb to the parasite. Competing against a healthy partner increased virulence by increasing development time. Interestingly, infected mosquitoes also fared better when paired with an infected competitor.

These results suggest that a high incidence, or prevalence, of parasitic infection in the population means that healthy larvae face less competition and do better than they would if they had to compete with healthy individuals. Infected individuals will also do better if there's a high prevalence of infection because they are more likely to compete against equally poor competitors. Thus, by ignoring the effects of competition, standard models underestimate the full costs of virulence—and, more important, miss a significant link between a parasite's prevalence in a population and its virulence.

Because the virulence of the parasite varies with its distribution in the population, this phenomenon may affect the population dynamics of parasite and host: infected hosts can't compete as well and take longer to develop, increasing the parasite's chances of transmission. The evolutionary implications of this relationship may be a selective pressure for host resistance to parasites. In traditional models, in low-virulence conditions, resistance is selected against because the cost of resistance reduces the fitness of the uninfected individual. But when low prevalence is associated with high virulence, the benefits of resistance would increase the individual's fitness. For an infected mosquito, at least, you stand a better chance of getting your wings and leaving the natal lagoon if more of your larval neighbors are infected too.

